# Human Papillomavirus Infection and Cervical Cytology Among Vulnerable Women in Rome, Italy

**DOI:** 10.3390/jcm15020817

**Published:** 2026-01-20

**Authors:** Eugenia Giuliani, Mauro Calandra, Maria Benevolo, Francesca Rollo, Francesca Sperati, Alessandra Sammali, Enrico Vizza, Edoardo Pescarmona, Valentina Laquintana, Aldo Morrone, Alessandra Latini, Maria Gabriella Donà

**Affiliations:** 1 STI/HIV Unit, San Gallicano Dermatological Institute IRCCS, 00144 Rome, Italy; eugenia.giuliani@ifo.it (E.G.); alessandra.latini@ifo.it (A.L.); mariagabriella.dona@ifo.it (M.G.D.); 2 Gynecology Department, IRCCS Regina Elena National Cancer Institute, 00144 Rome, Italy; mauro.calandra21@gmail.com (M.C.); enrico.vizza@ifo.it (E.V.); 3 Pathology Department, IRCCS Regina Elena National Cancer Institute, 00144 Rome, Italy; maria.benevolo@ifo.it (M.B.); edoardo.pescarmona@ifo.it (E.P.); valentina.laquintana@ifo.it (V.L.); 4 UOSD Clinical Trial Center, Biostatistics and Bioinformatics, Scientific Direction, San Gallicano Dermatological Institute IRCCS, 00144 Rome, Italy; francesca.sperati@ifo.it; 5 Scientific Direction, San Gallicano Dermatological Institute IRCCS, 00144 Rome, Italy; alessandra.sammali@ifo.it; 6 San Gallicano Dermatological Institute IRCCS, 00144 Rome, Italy; aldomorrone54@gmail.com

**Keywords:** human papillomavirus, sexually transmitted diseases, HPV, vaccines

## Abstract

**Background**: Vaccination against human papillomaviruses (HPVs) and cervical cancer screening represent effective tools for preventing this neoplasia, but access to health services is limited for vulnerable women. We investigated prevalence of high-risk HPV and abnormal cervical cytology, as well as knowledge about HPV and the HPV vaccine, among homeless and migrant women in Rome, Italy. **Methods**: Cytologic samples in PreservCyt (Hologic) were employed for liquid-based cytology (ThinPrep Processor 5000, Hologic) and high-risk HPV DNA testing (Xpert HPV assay, Cepheid). Socio-demographic data, anamnestic, and behavioral data were retrieved from electronic archives. A questionnaire was employed to assess knowledge about HPV and HPV vaccination. **Results**: A total of 134 women were included (median age: 43 years; interquartile range, IQR: 34–50), mostly coming from Central–South America (69, 51.5%). Of the 127 cytologic specimens collected, one (0.8%) was invalid for the HPV test and five (3.9%) were unsatisfactory for the morphological evaluation. High-risk HPV positivity was found in 18 women of the 126 women with a valid HPV test (14.3%). A total of 10 women of the 122 women with an adequate cytology (8.2%) had abnormal cytology. Overall, 57/134 women (42.6%) had never heard of HPV or were unsure about it. Only 29 of the 77 women who had heard of HPV (37.7%) knew of the HPV vaccine, and only 2 had been vaccinated in the entire study group (1.5%). **Conclusions**: Tailored preventive strategies and comprehensive information campaigns should be developed and implemented to enhance awareness of HPV infection and actively promote vaccination among women in vulnerable groups.

## 1. Introduction

Human papillomavirus (HPV) infection is the most common sexually transmitted viral infection worldwide. Some mucosal HPV genotypes, mainly HPVs 6 and 11, cause benign lesions (anogenital warts), while others, classified as high-risk (i.e., HPV16, 18, 31, 33, 35, 39, 45, 51, 52, 56, 58, 59) [1], are involved in the development of squamous cell carcinomas of the anogenital tract and the head and neck region [2]. HPV-related cancers develop when a persistent HPV infection is established, a condition that only occurs in a small proportion of cases. Most HPV infections, in fact, are transient, as the immune system is typically able to clear the virus effectively. Nevertheless, acquiring HPV, like other sexually transmitted infections, can significantly affect physical, psychological, and sexual well-being and HPV-positivity can also lead to loneliness, stigmatization, and social exclusion [3,4,5,6,7,8], particularly when high-risk genotypes associated with cancer are involved. Consequently, HPV acquisition may negatively influence overall quality of life, irrespective of the development of HPV-associated lesions.

Cervical cancer represents the HPV-associated malignancy with the highest incidence, ranking eighth worldwide in terms of incident case numbers [2]. Thanks to vaccination and screening, cervical cancer is a preventable disease. The nonavalent vaccine not only prevents infection by low-risk types 6 and 11 but also protects against high-risk types responsible for over 90% of cervical cancer cases (HPV 16, 18, 31, 33, 45, 52, and 58). In addition to primary prevention, secondary prevention strategies, based on HPV testing and/or the Pap test, are also highly effective, leading to a significant reduction in cervical cancer incidence and related mortality. Although these tools are available in high-income countries, like Italy, women belonging to so-called vulnerable populations, such as homeless women and migrants, suffer from reduced access to social and health services, including vaccination, screening, diagnosis, and treatment of HPV-related lesions. Factors such as social exclusion, cultural barriers, and disadvantaged socio-economic conditions can limit access to healthcare for particularly vulnerable female populations [9]. Migrant women often have an inadequate knowledge of HPV infection and cervical cancer risk factors, may perceive it as inappropriate to undergo a gynecological visit, or may experience discomfort or embarrassment when interacting with male healthcare providers. Therefore, there is an urgent need to overcome these barriers, increase awareness, and improve participation of vulnerable women in prevention programs.

The increase in migration flows observed in recent years has led to growing interest in the health of migrant populations. Official data show that as of mid-2024, 52% of the approximately 94 million immigrants living in Europe are women, most of them of reproductive age [10]. The risk of developing cervical cancer, as well as of delayed diagnosis and poor outcomes, is significantly increased among migrant women from low- and middle-income countries, where HPV prevalence is generally higher and where there are no organized screening programs. Even when they relocate to countries with well-organized screening programs, lack of information, few female healthcare providers, language barriers, and emotional factors like fear and embarrassment may represent common obstacles to screening access among migrant women in Europe [11]. Besides individual-level factors, system-level factors may also affect cervical cancer screening participation among migrant women. Screening policies and organizational aspects in the residing country, for instance, may play a role in limiting access to screening services [9]. The reduced screening participation rate ultimately results in migrant women being disproportionately affected by cervical cancer. A study conducted in southern Italy using cancer registry data showed increased proportions of cervical cancer cases among migrant women compared to non-migrant women [12].

During the pandemic, in order to carry out prevention and control activities for COVID-19, the San Gallicano Dermatological Institute IRCCS (Rome, Italy) began a collaboration with the homeless support center Binario 95 (Rome, Italy) and the Mother of Mercy Outpatient Clinic, run by the Apostolic Almonry of Vatican City, which offers free healthcare to those living in situations of indigence, marginalization, or difficulty. In May 2022, to build on the activities initiated during the pandemic, the same institute signed the “Health for All” memorandum of understanding to promote healthcare interventions for vulnerable people. As part of this collaboration, gynecological visits and cervical cancer screening were introduced among the health services offered by Binario 95 and the Mother of Mercy Outpatient Clinic. The aim of this study was to assess the prevalence of high-risk HPV infection and abnormal cervical cytology among the women assisted over the 12-month duration of the “Health for All” memorandum. Their knowledge of HPV infection and the HPV vaccine was also investigated.

## 2. Materials and Methods

### 2.1. Study Population

The study included women who underwent a gynecological visit and/or cervical cancer screening at Binario 95 or the Mother of Mercy Outpatient Clinic from May 2022 to April 2023. PAP tests and HPV tests were performed at the Pathology Department of the IRCCS Regina Elena National Cancer Institute (Rome, Italy). Socio-demographic data, PAP test and HPV test results were retrieved from the electronic archives of this department, whereas anamnestic and behavioral data were retrieved from questionnaires administered by the gynecologist prior to the consultation. The level of knowledge about HPV and HPV vaccination was also investigated through face-to-face interviews conducted by the gynecologist. When women were not able to understand and communicate in Italian, the gynecologist communicated in English or requested the presence of a French interpreter when necessary.

### 2.2. Cervical Cytology

Cervical samples were obtained using an Ayre spatula for esocervical cell collection and a cytobrush for endocervical cell collection. Cells were then dispersed in PreservCyt (Hologic, Pomezia, Italy) and used for liquid-based cytology using a ThinPrep Processor 5000 (Hologic). Cytologic slides were stained with Papanicolau staining and interpreted by an experienced cytologist, blinded to the HPV test result, following the guidelines of the Bethesda System [13].

### 2.3. High-Risk HPV Testing

High-risk HPV test was performed employing the Xpert^®^ HPV (Cepheid, Inc., Sunnyvale, CA, USA), using 1 mL of the cervical sample prior to processing the sample for cytology. The Xpert test is a qualitative test that detects 14 high-risk HPVs targeting an 80–150 bp fragment in the E6/E7 region. The test result is provided with concurrent partial genotyping, based on five different probes: Probe 1—HPV16; Probe 2–HPV18/45; Probe 3—HPV31/33/35/52/58; Probe 4—HPV51/59; Probe 5—HPV39/56/66/68. While positivity for Probe 1 and Probe 2 is provided individually, positivity for any of the other three probes is provided collectively, i.e., as positivity for “other high-risk HPVs”. Samples lacking sample adequacy control were considered as invalid by the analysis software.

### 2.4. Statistical Analyses

Descriptive statistics were calculated for all variables of interest: continuous variables were reported with their median values and interquartile ranges, and categorical variables as absolute frequencies and percentages. Univariate logistic regression models were applied to identify variables that might have played a role in the risk of high-risk HPV infection. All statistical analyses were performed using SPSS statistical software version 29 (SPSS Inc., Chicago, IL, USA).

## 3. Results

### 3.1. Study Population

During the study period, 134 women underwent a gynecological visit (median age: 43 years; interquartile range; IQR: 34–50). These women all were in a condition of frailty, e.g., unstable employment or inadequate socio-economic resources, no permanent housing, or substance abuse. Their median number of lifetime partners was three (IQR: 2–5) and the median age at first sexual intercourse was 18 years (IQR: 17–19). The other relevant socio-demographic, behavioral, and clinical characteristics are reported in Table 1. Most of the women were migrants, i.e., foreign-born, either in a regular situation or waiting for documentation. They mostly came from Central–South America (69; 51.5%), followed by European countries (45; 33.6%), specifically Ukraine, Poland, Romania, Moldavia, and Albania, besides Italy. Women coming from Africa and Asia were less represented. The majority of the women had completed high school (70; 52.2%). Sixty women (44.8%) reported to have no stable partner. Thirteen women (9.7%) did not have a permanent housing situation. Ninety-seven women (72.4%) had at least one child (up to nine). Thirty women (22.4%) had at least one spontaneous abortion, while 27 (20.1%) reported at least one voluntary pregnancy interruption (up to seven). Concerning lifestyle habits, 25 women (18.7%) were current smokers, and 17 (12.8%) were either current or past drug users. Six women (4.5%) declared that they had received a diagnosis of anogenital warts in the past. Forty-three women (32.1%) had never undergone a PAP test, and eleven (8.2%) were uncertain whether they had or not.

### 3.2. Cervical Cytology and HPV Test Results

Of the 134 women who underwent the gynecological visit, 7 (5.2%) did not undergo cervical sampling. Of the 127 cytological samples collected, 5 were inadequate for morphological interpretation (3.9%). The large majority of the 122 adequate samples (112, 91.8%) were classified as negative for intraepithelial lesion or malignancy (NILM). Two cases (1.6%) were classified as atypical squamous cells of undetermined significance (ASC-US), and eight (6.6%) were low-grade squamous intraepithelial lesions (LSILs). No cases of high-grade squamous intraepithelial lesion (HSIL) or squamous cell carcinoma (SCC) were observed. All 127 cervical samples except 1 tested valid at the HPV test. Of the 126 samples with a valid result, 18 (14.3%) tested positive for high-risk HPV. Specifically, four (3.2%) were HPV16-positive, nine (7.1%) were positive for Probe 3 only, two (1.6%) for Probe 4 only, and three (2.4%) for two different probes (Table 2).

The highest prevalence of high-risk HPV infection was found among Asian women (2/5, 40.0%), although they were very scarcely represented, followed by women coming from Central–South America (11/67, 16.4%) and Europe (5/41, 12.2%). No positive cases were observed among women from Africa (0/13, 0.0%).

Univariate logistic regression models did not identify any statistically significant predictors of high-risk HPV infection. Therefore, multivariate logistic regression models could not be run.

### 3.3. Knowledge of HPV and HPV Vaccine

Data on knowledge of HPV infection and the HPV vaccine are shown in Table 3. A significant proportion of the 134 women had never heard of HPV (38, 28.4%) or were unsure whether they had heard of this virus (19, 14.2%), with large differences according to country of origin. The large majority of the women from Central–South America had heard of HPV (56/69, 81.2%), with less than half of European women having heard of it (19/45, 42.2%). The proportion went further down among those coming from Africa (1/14, 7.1%) and Asia (1/6, 16.7%).

Of the 77 women who reported to have heard of HPV, 62 (80.5%) knew that HPV infection is sexually transmitted, and this proportion was the lowest among Central–South American women (43/56, 76.8%). Overall, the proportion of those who were aware of HPV role in cancer development was 72.7%, and the proportion of those who knew about the availability of an HPV vaccine was substantially lower (37.7%). Most of European women knew of the vaccine (13/19, 68.4%), while only a quarter of women from Central–South America were aware of this preventive opportunity (14/56, 25.0%). Only two women in the overall study group (1.5%) had been vaccinated. More than half of the 132 unvaccinated women (68, 51.5%) declared to be willing to be vaccinated once informed about the availability of the HPV vaccine, but a significant proportion were unsure about it (52, 39.4%). African women were the least inclined to vaccination (11/14, 78.6% were either contrary or unsure), while willingness to be vaccinated was the highest among Central–South American women (41/68, 60.3%).

## 4. Discussion

Migrant and homeless women represent fragile groups of women. Because of their socio-economic conditions, or cultural beliefs and societal norms, as well as a scarce level of knowledge about HPV and HPV-related diseases, they suffer from notable disparities in terms of health status, due to limited access to vaccination, screening, and treatments [9]. The present study aimed to evaluate the prevalence of cervical high-risk HPV infection and cytologic abnormalities in fragile women living in the city of Rome. From our results, a high-risk HPV prevalence of approximately 14% emerged. This prevalence exceeds the 8% reported among women participating in cervical cancer screening programs in Italy [14]. However, a direct comparison is not entirely appropriate, as HPV prevalence is strongly influenced by differences in age distribution, as well as in cytological abnormality prevalence. Considering only women with negative cytology, high-risk HPV prevalence among women in the present study is approximately 8% and overlaps with that found in women participating in Italian screening programs (i.e., 7% in the study by Giorgi Rossi et al., 2011) [15]. Other studies have reported widely varying rates of HPV prevalence among migrant women living in Italy. This ranges between 7% for regular migrants attending screening programs in Northern Italy [16] and almost 40% in migrant women enrolled in three prospective studies conducted in Southern Italy [17]. A more recent study conducted in Milan on over 500 migrant women found a positivity rate of about 24% [18]. This variability depends on several factors, including age, cervical cytology findings, and country of origin. Similarly to the study by Tornesello et al., 2014 [17], we observed the highest prevalence of high-risk HPVs in women from Asia, although only a minimal number of Asian women was included in our investigation. HPV prevalence, as well as genotype distribution, in migrant women may depend on the background prevalence in their country of origin. Migrant women who have recently relocated may still carry HPV genotypes that are more prevalent in their country of origin, particularly if they were exposed prior to migration. Unfortunately, we lack data on the time since arrival in Italy, which may be a contributing factor to HPV prevalence. Over time, the prevalence and type distribution may in fact shift to reflect patterns more typical of the resident population.

Given that the HPV test used does not provide full genotyping, data on HPV type-specific prevalence were not available. Positivity for Probe 3, which includes HPV31/33/35/52/58, was the most frequent finding, followed by positivity for HPV16, usually the most frequent high-risk genotype in all geographic areas [19]. The distribution of the other HPV genotypes causing genital infection may vary by ethnicity, with global surveys highlighting significant geographic variability.

In our study, approximately 8% of the women had abnormal cytology. This finding might seem surprising, given that around 40% of the women included in the present investigation had never undergone a PAP test before or were uncertain if they ever did. A recent meta-analysis evidenced a significantly lower participation rate in cervical cancer screening among migrant women compared with native women, especially in the case of migrant women coming from North Africa and Sub-Saharan Africa [20]. Based on the 18 included studies, a mean participation rate of 19.1% for migrant populations vs. 62.3% for native women emerged from this meta-analysis. For these potentially marginalized communities, it thus remains difficult to meet the WHO Global Strategy for the Elimination of Cervical Cancer by 2030, which is based on three core pillars: 90% of girls vaccinated by age 15, 70% of women screened with an HPV test by ages 35 and 45, and 90% of women with precancerous lesions or cancer receiving treatment [21]. Several studies have shown that migrant women are less likely to participate in organized programs for cervical cancer screening also in Italy, although some regional variations are observed [16,22,23,24]. In the Prato province, only 11% of the migrant women invited to participate adhered to screening, compared with over 50% of the Italian women [22], while differences were less prominent in other studies. Campari et al., who analyzed data from seven screening centers located in Northern Italy, reported a participation rate of 43.6% for regular immigrants and 52.2% for Italy-born women [16]. Battagello et al., consistently observed lower screening attendance among migrant women in the Veneto region, with a certain grade of variability according to the country of origin [23]. Women coming from Central/Southern America and Asia showed the lowest participation rates (less than 40%).

The adoption of innovative approaches, including self-sampling, warrants consideration to increase screening uptake also among migrant women. A meta-analysis demonstrated that self-sampling nearly doubles the probability of screening uptake compared with sampling by a clinician, also in under-screened populations [25]. Self-sampling procedures appeared acceptable and were preferred over clinician-collected samples by almost 70% of women in the pooled analysis. Notably, non-attender women preferred self-sampling to clinician-based sampling, highlighting the potential of the former strategy to engage never-screened women. In addition to providing the self-sampling option, screening in mobile units, the provision of interpreter services and cultural mediators, native-language education materials for clear communication on screening policy and other available preventive strategies may help raise awareness and facilitate access to prevention services [26,27]. Specific strategies to encourage vulnerable groups to participate in cervical cancer screening programs are already in place in some countries. In Italy, where a national screening program exists, these strategies involve sending invitation letters; however, this approach cannot be applied to all vulnerable women [28]. Currently, a European Union-funded project involving 10 countries, including Italy, is specifically pursuing the aim of improving cervical cancer screening among vulnerable and underserved groups [29]. In strict collaboration with these women, healthcare professionals, and decision makers, this project has identified both common and country-specific barriers to cervical cancer screening [30]. Providers’ lack of cultural and social sensitivity towards vulnerable women has emerged as a barrier in Italy, as well as in other countries. Psychological barriers, linked to women’s fear, shame, stigma, and lack of knowledge about preventive healthcare have been identified as further barriers across participating countries. Together with facilitators, these barriers represent a starting point for developing future interventions to improve cervical cancer screening among vulnerable women, thus contributing to the achievement of the WHO 2030 target of >70% of women screened for cervical cancer [21]. These strategies include community outreach, education, and system improvements. Nonetheless, the creation of tailored approaches appears pivotal, given the heterogeneity of vulnerable subgroups, e.g., in terms of their cultural background and life situation, as well as variability in local healthcare systems.

Most of the women included in this investigation had heard of HPV, but the proportion was extremely low for African and Asian women. However, awareness of HPV did not always imply knowledge of its sexual transmission or its involvement in cancer development. Notably, most of the women ignored the existence of the HPV vaccine or were unsure about it, and only 1.5% of the overall group had been vaccinated. According to a very recent meta-analysis, the pooled estimate of HPV vaccine uptake in migrant females is 23.0% [31]. A variety of factors emerged as negatively affecting the uptake, such as language barriers between migrant women and healthcare providers, cultural and religious beliefs, concern around vaccine safety, a low level of knowledge about HPV infection and vaccination, and the high cost of the vaccine. Given the barriers that emerged from this meta-analysis, a number of different approaches could help increase HPV vaccine uptake among migrant women. There is a need to develop effective health education and communication programs, while also addressing accessibility issues, for instance through mobile clinics or walk-in centers.

Several limitations may be acknowledged for this study: the small study group (the low participation in the screening program can also be explained by the scarce awareness of HPV infection and associated diseases); a possible self-selection bias (even among women in fragile conditions, those with a higher worry for their health status adhered to the program); the heterogeneity of the population in terms of country of origin; lack of information on the time since arrival in Italy for the migrant women. Finally, this program was only conducted in the city of Rome and may have “captured” diverse populations of fragile women compared to other Italian cities.

## 5. Conclusions

This investigation found that fragile women, including mainly migrants but also women lacking a permanent housing, have a high prevalence of high-risk HPV cervical infection. Nonetheless, the frequency of cytological abnormalities was in line with that observed for Italian women attending screening programs. Our findings regarding HPV knowledge, and especially vaccine uptake prompt us to implement initiatives to promote tailored information campaigns and vaccination among migrant women.

## Figures and Tables

**Table 1:** Socio-demographic, behavioral, and clinical characteristics of the 134 women included in the study.

Variables	n	%
*Geographic origin*		
African	14	10.4
Asian	6	4.5
European	45	33.6
Central–South American	69	51.5
*Education*		
Elementary	9	6.7
Middle school	31	23.1
High school	70	52.2
University	24	18.0
*Marital status*		
Married	33	24.6
Divorced	12	8.9
Widowed	2	1.5
Not married (single or cohabiting)	87	65.0
*Stable relationship*		
No	60	44.8
Yes	74	55.2
*Housing situation*		
Permanent housing	112	83.6
Accommodation center	6	4.5
Refugee center	4	3.0
Homeless shelter	3	2.2
Not available	9	6.7
*No. children*		
0	37	27.6
1	33	24.6
2	42	31.4
>3	22	16.4
*No. spontaneous abortions*		
0	53	39.5
≥1	30	22.4
Not available	51	38.1
*No. voluntary pregnancy interruptions*		
0	107	79.9
≥1	27	20.1
*Contraceptive method*		
None	110	82.1
Condom	15	11.3
Intrauterine device	3	2.2
Oral	3	2.2
Other hormonal	3	2.2
*Smoking*		
Never	105	78.3
Former	4	3.0
Current	25	18.7
*Drug consumption*		
Never	105	78.3
Former	15	11.3
Current	2	1.5
Not available	12	8.9
*Anogenital warts in the past*		
No	119	88.8
Yes	6	4.5
Unsure	9	6.7
*PAP test performed in the past*		
Never	43	32.1
Yes	80	59.7
Unsure	11	8.2

**Table 2:** High-risk HPV test results for the 126 women with a valid result.

High-Risk HPV Test Result	n (%)
High-risk HPV-negative	108 (85.7)
HPV16	4 (3.2)
HPV31/33/35/52/58 (Probe 3)	9 (7.1)
HPV31/33/35/52/58 (Probe 3) and HPV39/56/66/68 (Probe 5)	2 (1.6)
HPV51/59 (Probe 4)	2 (1.6)
HPV51/59 (Probe 4) and HPV39/56/66/68 (Probe 5)	1 (0.8)

**Table 3:** Knowledge of HPV infection and the HPV vaccine among the 134 women included in the study.

Question	n (%)
*Heard of HPV*	
No	38 (28.4)
Yes	77 (57.4)
Unsure	19 (14.2)
Total	134 (100.0)
*HPV as sexually transmitted*	
No	4 (5.2)
Yes	62 (80.5)
Unsure	11 (14.3)
Total	77 (100.0)
*HPV and cancer*	
No	2 (2.6)
Yes	56 (72.7)
Unsure	19 (24.7)
Total	77 (100.0)
*Heard of HPV vaccine*	
No	26 (33.8)
Yes	29 (37.7)
Unsure	22 (28.5)
Total	77 (100.0)
*Willing to be vaccinated against HPV* ^a^	
No	12 (9.1)
Yes	68 (51.5)
Unsure	52 (39.4)
Total	132 (100.0)

^a^ the two vaccinated women were excluded.

## Data Availability

The datasets analyzed during the current study are available from the corresponding author on reasonable request.

## Abbreviations

The following abbreviations are used in this manuscript:

HPV	Human papillomavirus
NILM	Negative for intraepithelial lesion or malignancy
ASC-US	Atypical squamous cells of undetermined significance
ASC-H	Atypical squamous cells—cannot exclude HSIL
LSIL	Low-grade squamous intraepithelial lesion
HSIL	High-grade squamous intraepithelial lesion
SCC	Squamous cell carcinoma
STI	Sexually transmitted infection
